# Correlates of cockroach nasal challenge responsiveness among sensitized urban children with asthma

**DOI:** 10.1016/j.jacig.2026.100684

**Published:** 2026-03-18

**Authors:** Lars E. Dunaway, Ricardo Da Silva Antunes, Anna Pomés, Matthew C. Altman, Jill Glesner, Basilin Benson, Kate Cho, Edward M. Zoratti, Robert A. Wood, Frederic F. Little, Jacqueline A. Pongracic, Gurjit K. Khurana Hershey, Michael G. Sherenian, Jeffrey M. Chambliss, Michelle A. Gill, Andrew H. Liu, Carin Lamm, Meyer Kattan, Leonard B. Bacharier, William J. Sheehan, Paula Busse, Alkis Togias, Lisa M. Wheatley, Patrice M. Becker, Cynthia M. Visness, William W. Busse, Alessandro Sette, Daniel J. Jackson

**Affiliations:** a Rho Federal Systems Division Inc, Durham, NC; b Center for Infectious Disease and Vaccine Research, La Jolla Institute for Immunology, La Jolla, Calif; c Basic Research, InBio, Charlottesville, Va; d Department of Allergy and Infectious Diseases, University of Washington, Seattle, Wash; e Systems Immunology Program, Benaroya Research Institute, Seattle, Wash; f Department of Medicine, Henry Ford Health System, Detroit, Mich; g Department of Pediatrics, Division of Allergy and Immunology, Johns Hopkins University School of Medicine, Baltimore, Md; h Boston University School of Medicine, Boston, Mass; i Ann & Robert H. Lurie Children’s Hospital of Chicago, Chicago, Ill; j Division of Asthma Research, Cincinnati Children’s Hospital Medical Center, Cincinnati, Ohio; k Department of Pediatrics, University of Texas Southwestern Medical Center, Dallas, Tex; l Department of Pediatrics, Washington University School of Medicine, St Louis, Mo; m Department of Allergy and Immunology, Children’s Hospital Colorado, University of Colorado School of Medicine, Aurora, Colo; n Division of Pediatric Pulmonology, Department of Pediatrics, College of Physicians and Surgeons, Columbia University, New York, NY; o Monroe Carell Jr Children’s Hospital at Vanderbilt University Medical Center, Nashville, Tenn; p Children’s National Health System, Washington, DC; q Icahn School of Medicine at Mount Sinai, New York, NY; r National Institutes of Health, National Institutes of Allergy and Infectious Diseases, Bethesda, Md; s Departments of Pediatrics and Medicine, University of Wisconsin School of Medicine and Public Health, Madison, Wis; t Department of Medicine, Division of Infectious Diseases and Global Public Health, University of California, San Diego, La Jolla, Calif

**Keywords:** Cockroach, NAC, CRITICAL, *Blattella germanica*, T cell, B cell, gene expression

## Abstract

**Background:**

The nasal allergen challenge (NAC) is a tool for evaluating upper airway allergic responses. In the CRITICAL study, NAC with cockroach allergen was used to confirm clinical reactivity in sensitized individuals from urban environments before enrollment onto a subcutaneous immunotherapy trial.

**Objective:**

Immunologic and transcriptomic predictors of NAC responsiveness were identified.

**Methods:**

NAC was performed in 103 participants. Clinical responses were assessed by the Total Nasal Symptom Score (TNSS) and sneeze score (TSNEEZ), which scale positively with symptom severity. Baseline immunologic markers—including skin prick test wheal size, cockroach-specific IgE, IgG, and IgG_4_, and T-cell responses were evaluated. Nasal lavage samples were analyzed for gene expression modules. Clinical trial registration: ClinicalTrials.gov NCT03541187.

**Results:**

Larger skin prick test wheal sizes were significantly associated with positive NAC outcomes (2.2 mm larger than negative NAC) and lower reactive doses (hazard ratio = 1.10). Cockroach (i6 extract)-specific IgE levels were inversely correlated with TNSS, while levels of IgE, IgG, and IgG_4_ specific to the extract used for therapy showed no association. Higher IL-10 T-cell responses were observed in those without reaction, while Bla g 5 and Bla g 9 dominance correlated negatively with TNSS and positively with TSNEEZ, respectively. Transcriptomic analysis revealed that higher expression of eosinophil- and neutrophil-associated modules and lower expression of type 1 interferon, macrophage, and epithelial barrier modules were linked to positive NAC responses.

**Conclusion:**

NAC responsiveness to cockroach extract is influenced by skin test reactivity, T- and B-cell regulation, and nasal gene expression. These findings highlight the role of both adaptive and innate immunity in allergic airway responses and suggest potential biomarkers for clinical reactivity.

## Introduction

The nasal allergen challenge (NAC) is an investigational procedure utilizing exposure of the nose to an environmental allergen extract to elicit an upper airway allergic response and to allow examination of the pathophysiology and mechanisms of this response.

In the CRITICAL study, a NAC to the cockroach extract used for therapy was performed at screening to confirm allergic symptoms to cockroach in the upper airway as a condition for enrollment and randomization into a clinical trial of cockroach subcutaneous immunotherapy in patients living in an urban environment who were sensitized to cockroach allergens.^1^ Cockroach extract was used as source of provoking allergens, as cockroaches produce important allergens for inhabitants of urban environments, which are a major risk factor for expression of asthma and its severity.

The response to a NAC varies from patient to patient, beginning with the dose of allergen required to elicit an allergic reaction of the nose. Although some trends were previously observed,^2^ no single biomarker could reliably predict clinical reactivity to NAC, underscoring the complexity of individual allergic response. The goal of this work was to identify the underlying allergic mechanisms that determine the severity of the allergic response of the nose to the allergen challenge. To accomplish this goal, a number of patient-associated variables were identified, including T-cell responses to the allergen and the following B-cell–dependent parameters: allergen-specific IgE, IgG, and IgG_4_. In addition, transcriptomic analyses were performed to identify molecular gene expression modules that were associated with the allergen challenge.^3^, ^4^, ^5^

We conducted the screening NAC with cockroach extract in 103 participants. Demographics for this sample are presented in Table I. The NAC outcomes evaluated in this report included each participant’s mean Total Nasal Symptom Score (TNSS) (see Table E1 in the Online Repository available at www.jaci-global.org), which positively relates to symptom burden, along with one of its components counting the number of sneezes, the sneeze score (TSNEEZ). A positive NAC was defined as having reached TNSS ≥ 6 or TSNEEZ = 3. The reactive dose, which is the dose that resulted in a positive NAC, was analyzed as well. Blood and nasal lavage samples, collected before NAC, allowed us to examine the association of B- and T-cell variables and baseline nasal gene expression patterns to the reaction to the NAC as well as to clinical patterns of response.

**Table I:** Demographic data of 103 subjects

Characteristic	No. (%) or median [IQR]
Site name	
Baltimore	6 (5.8)
Boston	4 (3.9)
Chicago	11 (11)
Cincinnati	26 (25)
Dallas	3 (2.9)
Denver	7 (6.8)
Henry Ford Health System	9 (8.7)
New York	18 (17)
New York Mt Sinai	5 (4.9)
St Louis	5 (4.9)
Washington, DC	9 (8.7)
Sex	
Female	45 (44)
Male	58 (56)
Age (years) at screening	12 [10, 14]
Race	
Black	66 (64)
Other	22 (21)
White	15 (15)
Ethnicity	
Hispanic or Latino	32 (31)
Not Hispanic or Latino	71 (69)

## Results and discussion

A larger skin prick test (SPT) wheal to German cockroach at baseline was associated with having a positive NAC (7.2 vs 5.0 mm; *P* < .001; Table II). Neither total IgE nor German cockroach–specific IgE (to the commercially available cockroach extract i6; ImmunoCAP) were associated with a positive NAC outcome. The cockroach SPT wheal size was also associated with the NAC reactive dose (hazard ratio = 1.10, *P* < .001; Table III), indicating that larger SPT wheal sizes relate to a higher rate of reaction during the challenge. The cockroach-specific IgE was negatively associated with symptom burden approximated by the mean TNSS (*R* = −0.23; *P* = .018).

**Table II:** German cockroach allergy biomarkers by NAC outcome at screening

Characteristic	No.	NAC outcome∗		Difference/ratio (95% CI)†	*P* value
		Negative	Positive		
Standard clinical assessments		n = 19	n = 84		
German cockroach SPT (mm)‡	103	5.0 (1.5)	7.2 (3.6)	2.2 (1.2, 3.3)	<.001
Total IgE (kU_A_/L)	102	472.88 (3.15)	539.02 (2.78)	1.14 (0.63, 2.06)	.65
German cockroach IgE (by cockroach extract i6 ImmunoCAP)	103	6.38 (4.93)	6.22 (5.75)	0.97 (0.42, 2.27)	.95
B-cell variables		n = 13	n = 82		
Spec IgE CR Ext (kU_A_/L)	95	2.95 (5.31)	5.13 (7.89)	1.74 (0.59, 5.13)	.30
Spec IgG_4_ CR Ext (mg_A_/L)	95	0.06 (1.20)	0.06 (2.58)	1.02 (0.65, 1.60)	.93
Spec IgE/IgG_4_ ratio	95	0.12 (4.47)	0.20 (6.31)	1.70 (0.65, 4.50)	.26
Spec IgG CR Ext-RO212 (mg_A_/L)	95	0.72 (2.93)	1.01 (3.69)	1.40 (0.70, 2.81)	.32
T-cell variables		n = 12	n = 74		
Total magnitude of responses	86	142.8 (1.8)	148.6 (2.7)	1.04 (0.68, 1.60)	.85
IL-4 response	86	40.8 (3.4)	45.1 (9.0)	1.11 (0.45, 2.71)	.82
IFN-γ response	86	24.7 (7.3)	23.9 (5.9)	0.97 (0.26, 3.58)	.96
IL-10 response	86	30.0 (2.2)	7.0 (7.9)	0.23 (0.12, 0.46)	<.001
Regulatory T-cell numbers	86	6,764.2 (1.8)	5,142.5 (3.5)	0.76 (0.48, 1.21)	.24

*CI,* Confidence interval; *CR Ext,* cockroach extract used for therapy; *Spec,* specific.

∗ Data are shown as geometric means (geometric standard deviations) for all assessments except German cockroach skin test,‡ which are reported as arithmetic means (arithmetic standard deviations).

† Data are shown as ratios of geometric means for all assessments except German cockroach SPT,‡ which are reported as differences in arithmetic means.

‡ Using study-specific cockroach extract.

**Table III:** Univariate analysis of B-cell variables and NAC outcomes

Characteristic	TNSS mean		TSNEEZ mean		Reactive dose		±NAC	
	Corr (95% CI)	*P*	Corr (95% CI)	*P*	Corr (95% CI)	*P*	Corr (95% CI)	*P*
Spec IgE CR Ext (kU_A_/L)	−0.17 (−0.37, 0.03)	.10	0.14 (−0.05, 0.34)	.15	1.10 (0.97, 1.24)	.12	1.74 (0.59, 5.13)	.30
Spec IgE to IgG CR Ext ratio	−0.16 (−0.36, 0.04)	.12	0.17 (−0.02, 0.37)	.08	1.05 (0.93, 1.19)	.45	1.24 (0.46, 3.34)	.65
Spec IgE to IgG_4_ CR Ext ratio	−0.18 (−0.38, 0.02)	.081	0.14 (−0.06, 0.34)	.17	1.09 (0.96, 1.24)	.20	1.70 (0.65, 4.50)	.26
Spec IgG CR Ext-RO212 (mg_A_/L)	−0.03 (−0.24, 0.17)	.74	−0.03 (−0.22, 0.17)	.80	1.15 (0.94, 1.42)	.18	1.40 (0.70, 2.81)	.32
Spec IgG_4_ CR Ext (mg_A_/L)	−0.02 (−0.22, 0.19)	.87	0.05 (−0.15, 0.25)	.64	1.11 (0.86, 1.44)	.42	1.02 (0.65, 1.60)	.93
Spec IgG_4_ to IgG CR Ext ratio	0.02 (−0.18, 0.23)	.83	0.06 (−0.14, 0.26)	.56	0.92 (0.75, 1.13)	.41	0.73 (0.41, 1.29)	.26
German cockroach IgE (kU_A_/L)	−0.23 (−0.42, −0.04)	.018	−0.01 (−0.21, 0.19)	.92	1.00 (0.88, 1.14)	>.99	0.97 (0.42, 2.26)	.95
German cockroach wheal size (mm)	0.08 (−0.12, 0.28)	.43	0.14 (−0.06, 0.33)	.17	1.10 (1.04, 1.17)	<.001	2.2 (1.2, 3.3)	<.001

*Corr,* Correlation; *CI,* confidence interval; *CR Ext,* cockroach extract used for therapy; *Spec,* specific.

Markers of B-cell reactivity specific to the German cockroach extract used in CRITICAL (Spec IgE Ext, Spec IgG_4_ Ext, and Spec IgG CR Ext-RO212), and their associated IgE/IgG_4_ ratio, were assessed as described in the Methods section in the Online Repository available at www.jaci-global.org. These markers were not associated with any of the NAC outcomes (Tables II and III).

T-cell responses were analyzed for magnitude, polarization, and dominance directed against 11 well-defined *Blattella germanica* (Bla g) allergens, utilizing activation-induced marker *ex vivo* assays as described in the Methods section in the Online Repository. A total of 14 T-cell variables were selected and analyzed in relation to clinical outcomes. IL-10 responses were higher in children with a negative NAC versus those with a positive NAC (30 vs 7; *P* < .001; Tables II and IV). In addition, a negative correlation was found between Bla g 5 dominance and the TNSS mean (*R* = −0.27, *P* = .013) as well as a positive correlation between Bla g 9 dominance and the TSNEEZ score (*R* = 0.23, *P* = .029).

**Table IV:** Univariate analysis of T-cell variables and NAC outcomes

Characteristic	TNSS mean		TSNEEZ mean		Reactive dose		±NAC	
	Corr (95% CI)	*P*	Corr (95% CI)	*P*	Corr (95% CI)	*P*	Corr (95% CI)	*P*
Bla g 5 (allergen dominance/relative %)	−0.27 (−0.47, −0.06)	.013	0.01 (−0.20, 0.22)	.94	0.99 (0.90, 1.09)	.85	2.13 (0.39, 11.6)	.35
Bla g 5 (allergen response)	−0.27 (−0.48, −0.06)	.013	0.02 (−0.19, 0.23)	.84	1.00 (0.92, 1.09)	.98	2.52 (0.39, 16.1)	.30
Bla g 7 (allergen dominance/relative %)	0.06 (−0.15, 0.28)	.56	0.05 (−0.16, 0.26)	.64	1.00 (0.91, 1.09)	.95	0.69 (0.10, 4.92)	.69
Bla g 7 (allergen response)	0.02 (−0.20, 0.23)	.88	0.06 (−0.15, 0.27)	.58	1.01 (0.93, 1.09)	.90	0.83 (0.10, 6.88)	.85
Bla g 9 (allergen dominance/relative %)	0.08 (−0.13, 0.30)	.44	0.23 (0.02, 0.44)	.029	1.08 (0.97, 1.19)	.15	2.33 (0.46, 11.9)	.28
Bla g 9 (allergen response)	0.03 (−0.18, 0.25)	.75	0.19 (−0.02, 0.39)	.081	1.06 (0.97, 1.15)	.18	2.59 (0.41, 16.5)	.29
IFN-γ (response)	−0.08 (−0.29, 0.14)	.48	0.02 (−0.19, 0.24)	.83	0.99 (0.88, 1.12)	.90	0.97 (0.26, 3.58)	.96
IFN-γ (T_H_1) (polarization)	0.02 (−0.19, 0.24)	.83	−0.01 (−0.22, 0.20)	.93	1.0 (0.88, 1.13)	.93	0.98 (0.30, 3.25)	.97
IL-10 (polarization)	−0.15 (−0.36, 0.07)	.17	−0.04 (−0.25, 0.17)	.70	0.91 (0.82, 1.01)	.088	0.23 (0.11, 0.47)	<.001
IL-10 (response)	−0.19 (−0.41, 0.02)	.073	−0.02 (−0.24, 0.19)	.83	0.93 (0.83, 1.03)	.15	0.23 (0.12, 0.46)	<.001
IL-4 (response)	−0.20 (−0.41, 0.02)	.068	0.06 (−0.15, 0.28)	.55	0.99 (0.87, 1.13)	.91	1.11 (0.45, 2.71)	.82
IL-4 (T_H_2) (polarization)	−0.19 (−0.41, 0.02)	.072	0.06 (−0.15, 0.27)	.59	0.97 (0.83, 1.13)	.68	1.00 (0.47, 2.13)	>.99
Total magnitude (response)	−0.16 (−0.38, 0.05)	.13	0.06 (−0.15, 0.27)	.57	1.01 (0.77, 1.31)	.96	1.04 (0.68, 1.60)	.85
Regulatory T-cell numbers	−0.12 (−0.33, 0.10)	.29	−0.09 (−0.30, 0.12)	.41	0.99 (0.81, 1.21)	.95	0.76 (0.48, 1.21)	.24

*Corr,* Correlation; *CI*, confidence interval.

Module gene expression from nasal lavage samples collected before the NAC were analyzed in relation to clinical outcomes using gene set enrichment analysis (aka GSEA) as described in the Methods section in the Online Repository. Higher expression (as assessed by GSEA normalized enrichment score) of an eosinophil-associated type 2 inflammation module (eos1) was significantly associated with a higher likelihood of having a positive NAC and was positively associated with higher TSNEEZ scores resultant from the NAC. Higher expression of a neutrophil-associated chemotaxis module (neut1) was also associated with a higher likelihood of having a positive NAC and was associated with higher TNSS scores resultant from the NAC. In contrast, lower expression of a type 1 interferon response module (m11), macrophage antimicrobial response modules (mac1 and mac2), and epithelial barrier modules (m13 and m5) were associated with having a positive NAC (false discovery rates < .05) (Table V).

**Table V:** Transcriptomic modules and NAC outcomes

Pathway	Cell association	Summary annotation	TNSS mean			TSNEEZ mean			Reactive dose			±NAC		
			NES	*P*	FDR	NES	*P*	FDR	NES	*P*	FDR	NES	*P*	FDR
CRITICAL_04	—	T2 inflammatory response	−1.62	.001	0.003	3.15	.001	0.002	−0.86	.956	1.000	1.26	.017	0.034
CRITICAL_09	—	Keratinization	−1.96	.001	0.003	−1.16	.114	0.148	1.67	.001	0.004	1.52	.001	0.004
CRITICAL_12	—	Histone modification	−0.87	.781	1.000	−1.89	.001	0.002	1.09	.267	0.415	1.31	.036	0.065
CRITICAL_16	—	Autophagy	−1.64	.001	0.003	−1.62	.002	0.004	0.82	.867	1.000	0.97	.524	0.611
eos1	Eosinophil	T2 inflammation	−1.95	.001	0.003	2.98	.001	0.002	1.46	.003	0.007	1.52	.002	0.006
eos3	Eosinophil	—												
Epithelium	Eosinophil activation, mucus hypersecretion	—	−1.68	.001	0.006	2.42	.001	0.002	0.96	.535	0.749	−1.56	.009	0.019
eos5	Eosinophil	Eicosanoid metabolism	−1.13	.260	0.482	2.43	.001	0.002	−1.05	.367	0.555	1.03	.407	0.496
epi1	Epithelium	EGFR signaling, cell–cell adhesion	−1.61	.001	0.003	1.29	.028	0.040	1.18	.112	0.196	−2.47	.001	0.004
mac1	Macrophage	Antigen processing and presentation	−0.83	.888	1.000	2.09	.001	0.002	−2.05	.001	0.004	−1.67	.001	0.004
mac2	Macrophage	Chemoattraction, cytotoxicity	1.53	.003	0.011	−2.12	.001	0.002	−0.97	.550	0.751	−1.61	.001	0.004
neut1	Neutrophil	Neutrophil chemotaxis	1.43	.007	0.019	−2.72	.001	0.002	1.64	.001	0.004	1.92	.001	0.004
neut5	Neutrophil	T1 interferon regulation	−0.82	.834	1.000	−2.76	.001	0.002	1.83	.001	0.004	−1.28	.069	0.110
unma11	—	T1 interferon response	−1.04	.371	0.629	−2.54	.001	0.002	1.00	.457	0.673	−2.25	.001	0.004
unma13	Epithelium	Extracellular matrix production	−1.75	.001	0.003	−1.20	.089	0.124	1.50	.001	0.004	−1.70	.001	0.004
unma27	Epithelium	TGFB/SMAD3 cell differentiation	−1.35	.074	0.147	0.82	.778	0.871	0.63	.988	1.000	0.78	.855	0.939
unma5	Epithelium	Cilia function, IL-33 response	−1.68	.001	0.003	1.31	.011	0.017	−1.36	.004	0.009	−2.73	.001	0.004

Transcriptomic measurements were assessed for 5 negative NAC participants and 80 positive NAC participants.

*FDR,* False discovery rate; *NES,* normalized enrichment score; *T1/2,* type 1/2; *TGFB/SMAD3,* transforming growth factor beta receptor 2/mothers against decapentaplegic homolog 3.

While SPT showed a good correlation with NAC outcomes, antibody levels to cockroach extract used for therapy alone (IgE, IgG, or IgG_4_) did not. The larger the wheal size, the earlier the response occurred during NAC (response to lower allergen doses). SPT is related to B-cell response to allergens and provides an estimate of an allergic reaction to the allergen to which the patient is sensitized. Discrepancies between SPT and nasal provocation tests have previously been reported. Some studies show no correlation between SPT and nasal or bronchial challenges in patients with asthma, or moderately positive correlations between SPT and nasal provocation tests among allergic rhinitis patients.^6^^,^^7^ In contrast, circulating serum-specific IgE is associated with systemic responses and may not accurately reflect an organ-specific hypersensitivity, such as the one measured in the nasal cavity mucosa during the NAC or the SPT that reflects IgE bonded to mast cells in the skin.^7^^,^^8^

Our findings regarding T cells suggest that IL-10 may blunt NAC responses, while enhanced Bla g 9 dominance promotes NAC responsiveness. These results align with observations that IL-10 plays a regulatory role in CD4^+^ T_H_2 cell function during allergic airway responses and in establishing allergen tolerance. The somewhat weak correlations here between T-cell parameters and NAC responsiveness is not surprising because a high heterogeneity of T-cell reactivity was observed among participants, both in magnitude of total cytokine response and the patterns of individual allergen dominance.^9^

The gene expression analyses indicated distinct immunologic patterns associated with clinical outcomes. Notably, aspects of both eosinophil-associated type 2 inflammation and neutrophil-associated chemotaxis indicated a higher likelihood of a positive reaction. Conversely, lower expression of antimicrobial, particularly interferon related response, and lower epithelial barrier responses indicated higher likelihood of a positive reaction. These results underscore the role of impaired epithelial and innate immune responses’ heightening susceptibility to allergen challenge; in addition, symptoms can relate to both eosinophil and neutrophil-associated patterns of inflammation. The result highlights the importance of the immune and epithelial state of the end organ (the respiratory mucosa) in contributing to variability in clinical reactivity and symptom severity, suggesting potential biomarkers and therapeutic targets for allergic responses.

One limitation to the results reported herein is that they are limited to sensitized individuals only. Further research may be warranted to evaluate these relationships in nonsensitized individuals, which could serve as a valuable negative control as well as potentially shedding more light onto the allergic mechanisms at play.

Among patients living in an urban environment who were sensitized to cockroach allergens, a limited number of B- and T-cell responses and gene transcription modules were shown to correlate with NAC outcomes. These analyses demonstrate many distinct immune responses to cockroach systemically in peripheral blood and in the airways.

**Key messages**

• SPT wheal size and IL-10 levels predict NAC response.
• German cockroach–specific IgE and allergen dominance patterns influence symptom severity scores.
• Nasal gene expression implicates impaired epithelial and innate immune responses to reactivity.

## Disclosure statement

Funded in whole or in part with federal funds from the 10.13039/100000060National Institute of Allergy and Infectious Diseases (NIAID), 10.13039/100000002National Institutes of Health (NIH), Department of Health and Human Services, under cooperative agreements 1UM1AI114271-01 and UM2AI117870. Additional support was provided by the National Center for Research Resources, National Center for Advancing Translational Sciences (NCATS), NIH, under grants NCRR/NIH U19 AI135731, and NCATS/NIH NCATS/NIH UL1TR001079, UL1TR001422, UL1TR000150, UL1 TR002535, UL1TR001876, and 5UL1TR001425-03. A.T., L.M.W., and P.M.B.’s coauthorship of this publication does not necessarily constitute endorsement by the NIAID, the NIH, or any other agency of the United States government.

Disclosure of potential conflict of interest: All authors, with the exception of M. Altman, A. Togias, P. Becker, and E. Zoratti, report grants from NIH/NIAID during the conduct of the study. M. Altman, B. Benson, P. Becker, A. Togias, and M. Gill have nothing to disclose outside the current report. J. Gern reports consulting fees from Arrowhead Pharmaceuticals and Meissa Vaccines Incorporated, stock options from Meissa Vaccines Incorporated, and two patents issued related to the methods to enhance the production of rhinoviruses, outside the current report. W. Sheehan reports grants from NIH/NIAID, consulting fees from Regeneron and Syneos Health, and research funding from Merck, outside the current report. W. Busse reports royalties in the form of personal payment from Elsevier, consulting fees in the form of personal payments from Sanofi, Regeneron, and GlaxoSmithKline, payment or honoraria for lectures, presentations, bureaus, etc, from Sanofi, Regeneron, and GlaxoSmithKline, and support for attending meetings or travel in the form of direct payment by the company from Sanofi, Regeneron, and GlaxoSmithKline, outside the current report. L. Bacharier reports royalties from Elsevier, consulting and/or personal fees from GlaxoSmithKline, Genentech/Novartis, DBV Technologies, Kinaset, OM Pharma, Recludix, Teva, AstraZeneca, WebMD/Medscape, Vectura, and American Board of Allergy and Immunology and consulting fees, honoraria, and medical writing fees from Sanofi/Regeneron and nonfinancial support from American Academy of Allergy Asthma & Immunology, outside the current report. G. K. K. Hershey reports grants from Adare during the study. C. Ober reports grants from NIH/NIAID during the conduct of the study, and personal fees from American Association of Asthma, Allergy & Immunology outside the current report. R. Wood reports grants from DBV, Aimmune, Regeneron, Genentech, Novaris, and Food Allergy Research and Education and royalties from UpToDate, outside the current report. D. Jackson reports grants or contracts from National Heart, Lung, and Blood Institute, NIH office of the director, and Regeneron, consulting fees from AbbVie, and participation on Upstream Bio, AstraZeneca, GlaxoSmithKline, Regeneron, Sanofi, Areteia, and Apogee, outside the current report. A. Liu reports during the conduct of the study grants from ResMed, AstraZeneca, Phadia/Thermo Fisher Scientific, Revenio, Labcorp, ResMed/Propeller Health, and Revenio, and grants from OM Pharma, outside the current report.
