# Supplementary material for: Correlates of cockroach nasal challenge responsiveness among sensitized urban children with asthma
Source: J Allergy Clin Immunol Glob. 2026 Mar 18;5(3):100684. doi: 10.1016/j.jacig.2026.100684 (PMC13089143; doi:10.1016/j.jacig.2026.100684)
Supplement: Supplementary Figs and Tables [file mmc1.docx]

**Correlates of cockroach nasal challenge responsiveness among sensitized urban children with asthma**

**Online Repository**

**Authors:**

Lars Dunaway, PhD^1^

Ricardo Da Silva Antunes^2^

Anna Pomés, PhD^3^

Matthew C. Altman, MD, MPhil^4^

Jill Glesner, BSc^3^

Basilin Benson^4^

Kate Cho, MStat^1^

Edward M. Zoratti, MD^5^

Robert A. Wood, MD^6^

Frederic F. Little, MD^7^

Jacqueline A. Pongracic, MD^8^

Gurjit K. Khurana Hershey, MD, PhD^9^

Michael G. Sherenian, MD^9^

Jeffrey M. Chambliss, MD^10^

Michelle A. Gill, MD, PhD^11^

Andrew H. Liu, MD^12^

Carin Lamm , MD^13^

Meyer Kattan, MD^13^

Leonard B. Bacharier, MD^14^

William Sheehan, MD^15^

Paula Busse, MD^16^

Alkis Togias, MD^17^

Lisa M. Wheatley, MD, MPH^17^

Patrice M. Becker, MD ^17^

Cynthia M. Visness, PhD, MPH^1^

William W. Busse, MD^18^

Alessandro Sette, PhD^19^

Daniel J. Jackson, MD^18^

**Affiliations:**

1. Rho Federal Systems Division, Inc, Durham, NC
2. Center for Infectious Disease and Vaccine Research, La Jolla Institute for Immunology (LJI), La Jolla, CA
3. Basic Research, InBio, Charlottesville, VA
4. Department of Allergy and Infectious Diseases, University of Washington, Seattle, WA; Systems Immunology Program, Benaroya Research Institute, Seattle, WA
5. Department of Medicine, Henry Ford Health System, Detroit, MI
6. Department of Pediatrics, Division of Allergy and Immunology, Johns Hopkins University School of Medicine, Baltimore, MD
7. Boston University School of Medicine, Boston, MA
8. Ann Robert H. Lurie Children’s Hospital of Chicago, Chicago, IL
9. Division of Asthma Research, Cincinnati Children's Hospital Medical Center, Cincinnati, OH
10. Department of Pediatrics, University of Texas Southwestern Medical Center, Dallas, TX
11. Department of Pediatrics, Washington University School of Medicine, St. Louis, MO
12. Department of Allergy and Immunology, Children’s Hospital Colorado, University of Colorado School of Medicine, Aurora, CO
13. Division of Pediatric Pulmonology, Department of Pediatrics, College of Physicians and Surgeons, Columbia University, New York, NY
14. Monroe Carell Jr. Children’s Hospital at Vanderbilt University Medical Center, Nashville, TN
15. Children’s National Health System, Washington, DC
16. Icahn School of Medicine at Mount Sinai, New York, NY
17. National Institutes of Health, National Institutes of Allergy and Infectious Diseases, Bethesda, MD
18. Departments of Pediatrics and Medicine, University of Wisconsin School of Medicine and Public Health, Madison, WI
19. Department of Medicine, Division of Infectious Diseases and Global Public Health, University of California, San Diego, La Jolla, CA

**Corresponding Author:**

Lars Dunaway, PhD

Rho Federal Systems Division, Inc, Durham, NC

2635 NC-54

Durham, NC 27713

Telephone: (919) 595 6603

Email: Lars_Dunaway@rhoworld.com

**Supplemental Methods**

**CRITICAL Study**

The Cockroach Immunotherapy in Children and Adolescents (CRITICAL) study (NCT03541187) is a randomized, double-blind, placebo-controlled, multicenter trial of non-standardized glycerinated German cockroach (*Blattella germanica*) allergenic extract or placebo treatment administered by subcutaneous injection. Children with persistent asthma ages 8-17 years were eligible for the trial if they had a positive skin test (wheal ≥ 3 mm larger than saline control) to German cockroach extract, German cockroach-specific IgE ≥ 0.35 kU_A_/L, and a positive nasal allergen challenge (NAC) to German cockroach. This paper reports on the 103 children screened for the trial and examines baseline immunologic and molecular factors related to the clinical responses to the NAC. The trial results have been published.^1^

## Characterization of study-specific German cockroach extract

German cockroach allergen extracts are not standardized and demonstrate widely varying and often low allergen content. The content of Bla g 1, Bla g 2 and Bla g 5 was measured by ELISA^2^ in commercially available extracts from various sources, and manufacture of a single lot from Stallergenes Greer Pharmaceuticals at their facility in San Diego (formerly Allermed) was commissioned because of the consistently measurable levels from this source of all 3 allergens, most notably Bla g 5. The allergen content of this extract (CR Ext) was extensively characterized using ELISA (InBio) and mass spectrometry (FDA). Based on stability testing, the extract was stored at -20°C and was the only extract being used during the CRITICAL study for allergen challenge, skin testing, specific antibody testing, and subcutaneous immunotherapy.

## Nasal lavage sample collection and processing

The nasal lavage was collected immediately after a nasal blow procedure as previously described.^3^ Holding the head level, the participant squeezed sterile sodium bicarbonate buffered normal saline from a 240 mL sinus rinse bottle (NeilMed Sinus Rinse) into the right nostril while a 50 mL falcon collection tube (tube 1) was held under the left nostril, until 15-20 mL fluid was collected from the left nostril. Then, occluding the right nostril, the participant gently blew the left nostril several times into a separate 50 mL collection tube (tube 2). Next, while the participant held the head backwards at approximately 45 degrees, 5-10 mL of saline from the sinus rinse bottle was squeezed into the right nostril, held in the nose for 2-3 seconds, and then the participant tilted the head forward and allowed the saline to drip into collection tube 2. The same procedure was repeated for the left nostril using a 3rd collection tube (tube 3) and tube 2. The entire procedure was then repeated again for both nostrils. The samples were kept on ice during and after the collection.

After collection, each tube was vortexed for 10 seconds and consolidated. Samples were centrifuged at 1300 x g at 4°C for 10 min. All but ~10 mL of supernatant was removed and discarded. Cell pellets were resuspended in the remaining supernatant. The resuspended pellets were passed through a 100-micron strainer (MACS SmartStrainer). The strained fluid was gently mixed by pipet to homogenize the sample. A volume of 1.3 mL was removed and set aside for slide generation, and the remainder was centrifuged at 1300 x g for 10 min at 4°C, the supernatant removed, and 1 mL of RNAprotect Saliva Reagent (Qiagen) added and the pellet resuspended. Samples were kept on ice and/or at 4°C during processing.

## Nasal allergen challenge

The NAC began with a 0 mcg/mL of Bla g 1 [negative control – 50% glycerin, 50% COCAS with 0.2% phenol (Stallergenes Greer, Lenoir, NC)] dose followed by up to 8 doses of increasing concentration (see Figure E1 in the Online Repository), until the participant reached a Total Nasal Symptom Score (TNSS) of 6 or more, had a sneezing score of 3, or their symptoms become intolerable (see Table E1 in the Online Repository). Collection of TNSS and peak expiratory flow (PEF) was done 10 minutes after each dose was administered. Serum samples were collected before each NAC and were analyzed for assessment of B cell reactivity and cockroach-specific T cell responses, respectively. Nasal lavage samples were collected both pre- and post-NAC. Pre-NAC lavage samples were collected within approximately 1 hour prior to the NAC, and post-NAC lavage samples were collected approximately 3 hours after the second dose of the NAC. However, due to the burden of sample processing, pre-NAC samples for the first 14 negative NACs were not processed or analyzed, leaving only 5 pre-NAC samples from negative NACs with analyzable data.

**Measurement of IgE, IgG, and IgG4 antibody levels**

Baseline serum IgE values for German cockroach (i6 ImmunoCAPs) were measured at a commercial lab (Viracor).

Cockroach-specific IgE, IgG and IgG4 antibody levels were measured using in-house allergen-extract (CR Ext) loaded streptavidin ImmunoCAPs by a Phadia 250 (Thermo Fisher Scientific, Portage, MI). IgE was also measured using i6 ImmunoCAPs (commercially available ImmunoCAPs loaded with cockroach extract). For in-house ImmunoCAP measurements, biotinylated cockroach extract was loaded and incubated on streptavidin ImmunoCAPs using a Phadia 100. ImmunoCAPs were transferred to a Phadia 250 where measurements of IgE, IgG and IgG4 were performed according to the manufacturer’s instructions. Sera were tested undiluted for IgE and 1:100 for IgG and IgG4. Sera with cockroach-specific IgE >100 KU_A_/L were re-tested at 1:5 dilution. The lower limits of quantification were 0.1 kU_A_/L for IgE and 0.07 mg/L for IgG4. Streptavidin ImmunoCAPs not loaded with cockroach extract were used to assess IgG non-specific binding. Because values of ≥ 5 mg/L were detected in 6% of the samples tested (and ≥ 2 mg/L in 35%), values of IgG bound to empty streptavidin ImmunoCAPs (RO212) were subtracted from the IgG values obtained with in-house prepared ImmunoCAPs. For log transformation of antibody data, values lower than the limit of detection were set to 0.05 kU/L for IgE and 0.035 mg/L for IgG4. If values of IgG to Ext minus IgG to the non-extract loaded streptavidin CAPs (RO212) (mg/L) were zero or negative, they were changed to the lowest value divided by 2 (0.015 mg/L). The steps performed to prepare the in-house cockroach-specific ImmunoCAPs are described in the following section.

**Optimization of in-house cockroach-specific ImmunoCAPs for antibody measurements**

**1. Allergen extract biotinylation**

Optimization of the cockroach extract (CR Ext) biotinylation was carried out by adding EZ-Link Sulfo-NHS-LC-Biotin (Thermo Scientific, Rockford, IL) at various folds of molar excess (20-40) to the extract as is or to extract that was previously dialyzed into PMSF or diluted with PBS. The biotinylated mix was incubated for 30 minutes and put over a pre-washed Zeba Desalt Spin Column (Thermo Scientific, Rockford, IL) 2 times and the concentration was determined either by Advanced Protein Assay (Cytoskeleton Inc., Denver, CO) or by the Pierce BCA Protein Assay (Thermo Scientific, Rockford, IL).

**2. Optimization of biotinylated extract loaded to the streptavidin ImmunoCAP**

To optimize the amount of the extract loaded per ImmunoCAP, streptavidin ImmunoCAPs (Thermo Fisher Scientific, Portage, MI) were loaded and incubated on the Phadia 100, with the biotinylated extract at 5-8 concentrations within the range 0.1-20 µg/CAP. Two human sera previously tested by ImmunoCAP for their allergen specificity (using 3 µg of extract/CAP) were selected and tested for binding to the extract at these amounts. Optimal amounts of extract loaded to the ImmunoCAPs were determined from the results.

**3. Stability study of allergen loaded into the ImmunoCAP.**

Biotinylated extract was loaded and incubated on streptavidin ImmunoCAPs at its optimized concentration. CAPS were then stored at 4°C until run with 3 allergen-specific sera three days afterwards. Results showed that the loaded ImmunoCAPs were stable at 4°C for up to 3 days.

**PBMC isolation**

Blood was collected from whole blood by using CPT tubes (approximately 8 mL for each CPT tube (BD Vacutainer CPT tube with sodium heparin BD 362753, BD Biosciences, Franklin Lakes, NJ, USA)), gently inverted, and immediately centrifuged. Peripheral blood mononuclear cells (PBMC) were collected by fine aspiration from under the plasma layer, suspended in fetal bovine serum (FBS) containing 10% (vol/vol) dimethyl sulfoxide (DMSO) and stored in each clinical facility at -80ºC immediately after processing and then shipped to the La Jolla Institute (LJI) and cryopreserved in liquid nitrogen until use. Quality controls (QC) were performed in each sample to ensure quality based on determination of cell viability by trypan blue upon thawing and assessment of cell number yield.

**Activation Induced Marker (AIM) assay**

Evaluations of T cell responses were performed based on previously described Activation Induced Marker (AIM) *ex vivo* assays^4, 5^ using peptides for the 11 major cockroach allergens. AIM assays were performed utilizing the CD154 (CD40L) surface marker, combined with intracellular cytokine staining (ICS) for detection of IL-4, IFN-γ, or IL-10. Individual cytokine signals were analyzed by flow cytometry by gating CD4^+^CD154^+^Cytokine^+^ cells as previously described.^5^ Individual peptides (were resuspended in DMSO at a final concentration of 40 mg/mL. The peptides were pooled, lyophilized, and the resulting pool of peptides for each allergen was resuspended to a final concentration of 1 mg/mL. Peripheral blood mononuclear cells (PBMC) were thawed and rested overnight, plated at 2 × 10^6^ cells per well in a 96-well plate. 12-18h later, cells were stimulated with sets of individual peptide pools (2 µg/mL) for different allergens, phorbol myristate acetate (PMA) and Ionomycin (Ion) (positive control), or medium alone with DMSO (negative control) in the presence of 1 µg/mL CD40 (Miltenyi Biotec, Auburn, CA, USA). Cells were incubated for 6 h, adding Brefeldin A (1 µg/mL) for the last 3 h. After the incubation, cells were labeled with anti-CD154, -CD137, -CD25, -CD127, -CD4, -CD3, CD8, -CD14, -CD19, and live/dead fixable viability dye (Life Technologies, San Diego, CA, USA). After staining and washing, cells were fixed and permeabilized for intracellular staining (ICS), which was performed with anti-CD154, -CD137, -IL-4, -IFNγ, and -IL-10 (BD Biosciences, Franklin Lakes, NJ, USA), and probed by flow cytometry using a BD LSR II flow cytometer.

**Assessments of T cell responses**

The cytokines IL-4, IFNγ, and IL-10 were included as representative of Th2, Th1, and Tr1/T Regulatory (Tregs) CD4 helper T cell subsets, respectively.^3, 6^ The phenotypic CD127 and CD25 Tregs markers were also included in the cytometry analysis panel and the several T cell parameters used to infer associations with NAC determined as described in the supporting information.

With the AIM/ICS methodology described above, we determined: 1) Individual cytokine response: IL-4, IFNγ or IL-10 production calculated by summing positive signal in CD154+ CD4+ T cells for all CR allergen pools of each participant and expressed as number of cells per 10^6^ of total CD4+ T cells; 2) Magnitude of response: Sum of total IL-4, IFNγ and IL-10 cytokine responses (Teff; Effector T cells); 3) Polarization of response: Percentage of the total cytokine response ascribed to each of the three cytokines; 4) Dominance of response: Percentage of the overall response ascribed to each of the eleven allergens for all the cohort combined or individually for each participant. 5) Treg numbers: Identified as CD4+CD137+CD154-expressing cells^4, 5^ and represented as number of cells per 10^6^ of total CD4+ T cells. Data were analyzed using FlowJo software (TreeStar, Ashland, OR, USA). T cell responses in this study included only donors where enough cells allowed for testing of all the allergens (n=68).

**Nasal transcriptome measurements**

Total RNA was isolated from pre-NAC nasal lavage cell pellets in RNAprotect Saliva Reagent (Qiagen). Samples were centrifuged for 10 minutes at 10,000g, supernatant was removed, and the cell pellet was resuspended in 350 μL RLT Buffer + 1% beta mercaptoethanol. The sample was vortexed for 1-2 seconds, sonicated for 30 seconds, spun through a Qiashredder column (Qiagen) and then extracted using the RNeasy MinElute spin column (Qiagen) following the manufacturer’s protocol. RNA quality was assessed using RNA 115 electrophoresis (Agilent) and NanoDrop 1000 (NanoDrop Products, Thermo Fisher Scientific).

Sequencing libraries were constructed from total RNA using SMART-Seq v4 Ultra Low Input RNA Kit (Takara) and clustered onto a flowcell using a cBOT amplification system with a HiSeq SR v4 Cluster Kit (Illumina). Single-read sequencing was carried out on a HiSeq2500 sequencer (Illumina), using a HiSeq SBS v4 Kit to generate 58-base reads.

Reads were processed using workflows managed on the Galaxy platform. Reads were trimmed by 1 base at the 3’ end, and then trimmed from both ends until base calls had a minimum quality score of at least 30 (Galaxy FASTQ Trimmer tool v1.0.0). FastqMcf (v1.1.2) was used to remove any remaining adapter sequence. To align the trimmed reads, we used the STAR aligner (v2.4.2a) with the GRCh38 reference genome and gene annotations from ensembl release 91. Gene counts were generated using HTSeq-count (v0.4.1). Quality metrics were compiled from PICARD (v1.134), FASTQC (v0.11.3), Samtools (v1.2), and HTSeq-count (v0.4.1). For quality control, samples were kept that had percent of reads aligned >75%, total aligned reads >1,000,000, and median CV coverage <0.75, to filter out low quality libraries. Genes were filtered to include those that had a trimmed mean of M values (TMM) normalization count of at least 1 in at least 10% of libraries and were classified as protein coding using BioMart. Counts were transformed to log2 counts per million along with observations level weights using voomWithQualityWeights from the limma R package. The final dataset included 134 nasal samples and 14925 genes. Sequencing was performed in 3 sample batches and a batch correction was done using limma 136 removeBatcheffects. Accession number GSE250580.

**Statistical Analyses**

For the German cockroach skin test clinical assessment, the arithmetic mean was calculated for the NAC outcome groups. The ratio of the means of the positive and negative responders was calculated. For all other assessments, geometric mean was calculated for the NAC outcome groups, along with a ratio of the means of the different NAC outcomes. The Satterthwaite method was used to calculate the 95% confidence interval and the p-value of the ratio for all clinical assessments.

The Spearman correlation was calculated between antibodies, wheal size and cytokines and clinical outcomes. An unadjusted Cox regression model was used to analyze the change in NAC reactive dose between treatment groups.

Due to the skewed distribution, IgE and IgG4 values were log transformed. Values lower than the limit of detection were set to positive values as indicated in the Online Repository. For log transformation of cytokine data, values lower than the limit of detection were set to 0.1.

Differentially expressed genes were identified by linear modeling using the kimma R package.^7^ Four primary models were run, the first compared expression by NAC outcome (positive vs negative). Each of the other models compared expression among all samples to TNSS, TSNEEZ, or reactive dose. Results of these models were assessed by Gene Set Enrichment Analysis (GSEA)^8, 9^ utilizing as gene sets our previously defined and annotated repertoire of nasal modules from the CRITICAL^1^ and the MUPPITS^3, 10^ studies. Multiple testing correction was performed on GSEA results using the Benjamini-Hochberg method.

**Supplementary References**

1. Zoratti E, Wood R, Pomés A, Da Silva Antunes R, Altman MC, Benson B, et al. A pediatric randomized, controlled trial of German cockroach subcutaneous immunotherapy. J Allergy Clin Immunol. 2024;154(3):735-44 e10.

2. Glesner J, Filep S, Vailes LD, Wunschmann S, Chapman MD, Birrueta G, et al. Allergen content in German cockroach extracts and sensitization profiles to a new expanded set of cockroach allergens determine in vitro extract potency for IgE reactivity. J Allergy Clin Immunol. 2019;143(4):1474-81 e8.

3. Altman MC, Gill MA, Whalen E, Babineau DC, Shao B, Liu AH, et al. Transcriptome networks identify mechanisms of viral and nonviral asthma exacerbations in children. Nat Immunol. 2019;20(5):637-51.

4. Bacher P, Heinrich F, Stervbo U, Nienen M, Vahldieck M, Iwert C, et al. Regulatory T Cell Specificity Directs Tolerance versus Allergy against Aeroantigens in Humans. Cell. 2016;167(4):1067-78 e16.

5. da Silva Antunes R, Sutherland A, Frazier A, Schulten V, Pomés A, Glesner J, et al. Heterogeneity of magnitude, allergen immunodominance, and cytokine polarization of cockroach allergen-specific T cell responses in allergic sensitized children. Clin Transl Allergy. 2021;11(8):e12073.

6. Schulten V, Westernberg L, Birrueta G, Sidney J, Paul S, Busse P, et al. Allergen and Epitope Targets of Mouse-Specific T Cell Responses in Allergy and Asthma. Front Immunol. 2018;9:235.

7. Dill-McFarland KA, Mitchell K, Batchu S, Segnitz RM, Benson B, Janczyk T, et al. Kimma: flexible linear mixed effects modeling with kinship covariance for RNA-seq data. Bioinformatics. 2023;39(5).

8. Mootha VK, Lindgren CM, Eriksson KF, Subramanian A, Sihag S, Lehar J, et al. PGC-1alpha-responsive genes involved in oxidative phosphorylation are coordinately downregulated in human diabetes. Nat Genet. 2003;34(3):267-73.

9. Subramanian A, Tamayo P, Mootha VK, Mukherjee S, Ebert BL, Gillette MA, et al. Gene set enrichment analysis: a knowledge-based approach for interpreting genome-wide expression profiles. Proc Natl Acad Sci U S A. 2005;102(43):15545-50.

10. Jackson DJ, Bacharier LB, Gergen PJ, Gagalis L, Calatroni A, Wellford S, et al. Mepolizumab for urban children with exacerbation-prone eosinophilic asthma in the USA (MUPPITS-2): a randomised, double-blind, placebo-controlled, parallel-group trial. Lancet. 2022;400(10351):502-11.

**Table E1.** TNSS Scoring

| **Category** | Measurement | **Points** |
| --- | --- | --- |
| **Sneezing**  **Sneeze Count = __** | none | 0 |
|  | 1-2 | 1 |
|  | 3-4 | 2 |
|  | 5 or more | 3 |
| **Runny nose** | None - my nose feels fine and not runny at all | 0 |
|  | Mild - my nose is a little runny; I might be able to blow a little snot or mucous out | 1 |
|  | Moderate - my nose is very runny; I could definitely blow out some snot or mucous | 2 |
|  | Severe - my nose is extremely runny; I could blow out a lot of snot or mucous | 3 |
| **Stuffy nose** | None - my nose feels fine; I am not at all stuffy or congested | 0 |
|  | Mild - my nose feels a little stuffy | 1 |
|  | Moderate - my nose feels very stuffy; it is hard to breathe through my nose | 2 |
|  | Severe - my nose is completely stuffy; I cannot even breathe through my nose | 3 |
| **Itchy Nose** | None - my nose does not itch at all | 0 |
|  | Mild - my nose feels a little itchy | 1 |
|  | Moderate - my nose feels very itchy | 2 |
|  | Severe - my nose is extremely itchy | 3 |
| **TNSS** | | /12 |

**Fig E1.** Dilution scheme for cockroach extract
